# Early oncological outcomes of delayed radical prostatectomy: A prospective, international, follow‐up analysis of the COVIDSurg‐Cancer study

**DOI:** 10.1002/bco2.433

**Published:** 2024-10-17

**Authors:** Arjun Nathan, Chuanyu Gao, Alexander Light, Cameron Alexander, Vinson Chan, Kevin Gallagher, Sinan Khadhouri, Kevin G. Byrnes, Michael Ng, Michael Walters, Terry Hughes, Rita J. Perry, Kelvin Okoth, Laura Magill, Thomas Pinkney, Yuhao Zhang, James Blackmur, Eric Etchill, Stanley Tang, Damián García Escudero, Alan McNeill, Krishna Narahari, Grant D. Stewart, Veeru Kasivisvanathan, COVIDSurg Collaborative

**Affiliations:** ^1^ Division of Surgery and Interventional Sciences University College London London UK; ^2^ British Urology Researchers in Surgical Training (BURST) Research Collaborative London UK; ^3^ Department of Surgery and Cancer Imperial College London London UK; ^4^ Department of Urology NHS Lothian Edinburgh UK; ^5^ Urology Department Royal Devon and Exeter NHS Foundation Trust Exeter UK; ^6^ Birmingham Centre for Observational and Prospective Studies Birmingham UK; ^7^ Stepping Hill Hospital, Stockport NHS Foundation Trust Stockport UK; ^8^ Department of Urology Addenbrooke's Hospital Cambridge UK; ^9^ Johns Hopkins Hospital Baltimore Maryland USA; ^10^ Hospital General Reina Sofía Murcia Spain; ^11^ University Hospital of Wales Cardiff and Vale University Health Board Cardiff UK; ^12^ Department of Surgery University of Cambridge Cambridge UK

**Keywords:** COVID, delay, prostate cancer, radical prostatectomy, surgery

## Abstract

**Objectives:**

The objective of this study is to compare the early oncological outcomes of delayed (>90 days) versus scheduled (≤90 days) radical prostatectomy (RP).

**Patients and methods:**

Patients with prostate cancer due to undergo surgery between March 2020 and June 2020 who were enrolled in the COVIDSurg‐Cancer international, observational study were prospectively followed up for 1 year. Time to surgery was defined as the difference between the operation date and the multi‐disciplinary team decision to offer surgery. The primary outcome was the positive surgical margin (PSM) rate. Biochemical recurrence (BCR), upgradation and upstaging were secondary oncological outcomes. The Independent *t*‐test and Mann Whitney *U* test were used to compare means between groups and regression models and were used to investigate factors associated with the primary outcome.

**Results:**

Four hundred seventy‐six (78.7%) patients underwent RP from 605 that were eligible. Three hundred seven (64.5%) patients underwent scheduled RP, and 169 (35.5%) underwent delayed RP. A small proportion of men (*n* = 35, 6.8%) did not undergo RP within the 1‐year follow‐up period. More men with high‐risk disease (72.8%) underwent scheduled RP compared to men with intermediate‐risk disease (60.2%) (*p* < 0.05). There was no statistically significant difference in the PSM rate between the two groups (*p* = 0.512). Delay in surgery was not associated with an increased PSM or BCR on univariable or multivariable analyses. There was statistically significantly greater upstaging (*p* < 0.05) in the delayed group but no difference in upgradation.

**Conclusion:**

High‐risk men were prioritised for surgery during the COVID‐19 pandemic. Our prospective data support previous retrospective, cancer‐registry evidence suggesting no adverse oncological impact after delaying RP across all risk groups. Our study is limited by the short follow‐up period, and therefore, longer term conclusions cannot be drawn.

## 1 INTRODUCTION

Radical prostatectomy (RP) is one of the treatment modalities indicated for the management of intermediate‐ to high‐risk localised prostate cancer (PCa).^1^ However, there is no international consensus regarding the timeframe in which RP should be performed. In the United Kingdom, cancer waiting time directives recommend that RP should be undertaken within 62 days from the date of initial cancer suspicion.^2^ Increasing the time between diagnosis and RP may give patients more time to consider an increasing number of available treatment modalities and reduce decision regret.^3^ Additionally, hospital services may benefit from increased flexibility in order to efficiently manage resources and waiting list pressures.

There is conflicting evidence as to whether oncological outcomes are negatively impacted by delaying RP. Previous studies report no adverse oncological implication when radical treatment is delayed, whilst others describe that a delay of approximately 3 months may result in inferior oncological outcomes, specifically in high‐risk disease.^4^, ^5^, ^6^, ^7^

The COVID‐19 pandemic significantly disrupted medical care across the world. The COVIDSurg Collaborative estimated that over 28 million operations were postponed or cancelled across an average 12‐week period during the first wave.^8^ The National Comprehensive Cancer Network suggested that all treatment for PCa could be delayed by up to 6 months during the pandemic.^9^ However, other expert panels suggested that high‐risk PCa treatment should be prioritised.^10^, ^11^

The lack of clear consensus regarding the timing of RP is due to the poor‐quality evidence available. Current studies are limited to single‐institution retrospective or national cancer‐registry database analyses with no prospective, patient‐specific data available. Using the COVIDSurg‐Cancer international cohort database, we report the global practice of RP during the initial pandemic, and 1‐year oncological outcomes of delayed RP.

## 2 METHODS

### 2.1 Study design

The COVIDSurg‐Cancer collaborative cohort study collected international, multi‐centre, patient‐level data on patients due to undergo cancer surgery during the COVID‐19 pandemic.^12^ The population at risk was defined as patients older than 18 years with a confirmed cancer diagnosis with a definitive recommendation from the multi‐disciplinary team (MDT) to offer surgery with curative intent.

### 2.2 Participants

From this COVIDSurg‐Cancer study, patients who were due to undergo RP for PCa between March 2020 and June 2020 were prospectively followed up for 1 year from the date of the MDT decision to operate. All centres that participated in the initial study were eligible for the follow‐up study.

### 2.3 Data variables

We defined a delay in RP as more than 90 days between the MDT decision to operate and the operation date based on the concerns raised by previous literature describing adverse outcomes associated with a delay to surgery between 75 and 90 days.^4^, ^5^ Patients in this group were categorised as the ‘delayed’ sub‐group. Patients who underwent RP within and inclusive of 90‐days were categorised as ‘scheduled’. Pre‐operative demographic and oncological data including prostate‐specific antigen (PSA), International Society of Urological Pathologists (ISUP) grade, T‐stage, European Association of Urology risk score and the use of pre‐operative neoadjuvant androgen deprivation therapy (ADT) were collected along with peri‐operative data and oncological follow‐up outcomes. Biochemical recurrence (BCR) was defined as a PSA > 0.2 ng/mL, upstaging was defined as an increase between clinical and pathological T‐score, and upgradation was defined as an increase between biopsy Gleason score and specimen Gleason score. Data were captured via the Research Electronic Data Capture (REDCap) system.^13^

### 2.4 Outcomes

The primary outcome was the positive surgical margin (PSM) rate. PSM was used as an early oncological surrogate indicator for longer term oncological outcomes.^14^, ^15^ BCR at 1 year, upstaging and upgradation were secondary outcomes.

### 2.5 Statistical analysis

Data analysis was undertaken using Stata version 15 (StataCorp LLC, College Station, TX, USA). Baseline patient characteristics for the scheduled and delayed RP groups were compared descriptively. Continuous data with a normal distribution were summarised using means (standard deviation) and compared using the Independent *t*‐test. Continuous data with a skewed distribution were summarised using median (inter‐quartile range) and compared using Mann–Whitney *U* test. Categorical data were summarised using number (%) and compared using the Chi‐squared test. Univariable and multivariable binary logistic regression models were used to determine the association between PSM (yes/no), BCR and pre‐specified independent predictors based on literature. Effect estimates were presented as odds ratios (OR) and associated 95% confidence interval (95% CI). In all analyses, a two‐tailed *p*‐value of <0.05 was considered statistically significant. Study data were collected and managed using REDCap electronic data capture tools hosted at University of Birmingham. REDCap is a secure, web‐based software platform designed to support data capture for research studies, providing (1) an intuitive interface for validated data capture; (2) audit trails for tracking data manipulation and export procedures; (3) automated export procedures for seamless data downloads to common statistical packages; and (4) procedures for data integration and interoperability with external sources.

## 3 RESULTS

### 3.1 Population

**FIGURE 1:**
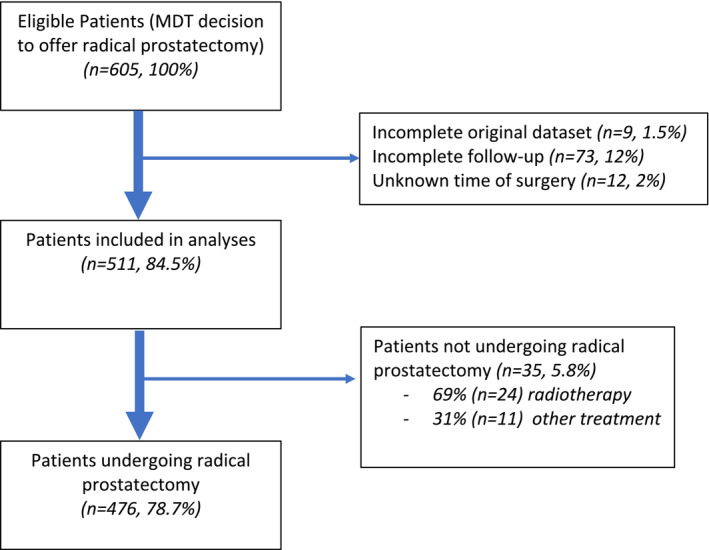
Flow diagram of patients included in the study. MDT, multi‐disciplinary team.

Figure 1 shows the cohort flow diagram. Five hundred eleven (84.5%) patients were included in the analysis from 605 eligible patients. Four hundred seventy‐six (78.7%) underwent RP, of which 307 (64.5%) were scheduled and 169 (35.5%) delayed. Twenty‐one (3.5%) patients had incomplete data, 73 (12.0%) were lost to follow‐up and 35 (5.8%) had an alternative treatment other than RP.

### 3.2 Demographics and pre‐operative data

More men with high‐risk disease (72.8%) underwent scheduled RP compared to men with intermediate‐risk disease (60.2%) (*p* < 0.05). There were no statistically significant differences between delayed and scheduled RP with regards to age; however, there was a greater proportion of men in the scheduled group who had a higher body mass index (BMI) (BMI > 25, 67% vs. 60%, *p* < 0.05), American Society of Anaesthesiology (ASA) score (ASA ≥ 3, 14% vs. 12%, *p* < 0.05) or Charlson co‐morbidity index (CCI) score (CCI ≥ 3, 63% vs. 51%, *p* < 0.05). There was no significant difference in the proportion of men that received neoadjuvant ADT between scheduled (2%, *n* = 7) and delayed surgery (2%, *n* = 3). Further pre‐operative oncological data are presented in Table 1.

**TABLE 1:** Demographic and pre‐operative data of patients who underwent surgery.

	Overall	Time to surgery		
		Scheduled ≤90 days	Delayed >90 days	*p*‐Value
*n* (%)	476	307	169	
Age (years) *n* (%)				0.474
<60	129 (27)	88 (28)	41 (24)	
60–69	247 (52)	156 (51)	91 (54)	
>70	100 (21)	63 (21)	37 (22)	
Body mass index *n* (%)				0.026
Underweight or normal (<18.5–24.9)	162 (34)	100 (33)	62 (37)	
Overweight or obese (>25)	305 (64)	205 (67)	100 (60)	
Missing	9 (2)	2 (1)	7 (4)	
ASA *n* (%)				0.001
1	103 (22)	48 (16)	55 (33)	
2	311 (65)	218 (71)	93 (55)	
≥3	62 (13)	41 (14)	21 (12)	
Charlson co‐morbidity index score *n* (%)				0.019
0 or 1	66 (14)	43 (14)	23 (13)	
2	130 (27)	70 (23)	60 (36)	
≥3	280 (59)	194 (63)	86 (51)	
PSA (ng/mL) (*n* = 460) median (IQR)	7.9 (5.5–12.1)	8.0 (5.6–12.0)	7.6 (5.6–12.4)	0.721
ISUP grade *n* (%)				0.017
1	54 (11)	35 (11)	19 (11)	
2	225 (47)	134 (44)	91 (54)	
3	98 (21)	67 (22)	31 (18)	
4	49 (10)	34 (11)	15 (9)	
5	36 (8)	31 (10)	5 (3)	
Missing	14 (3)	6 (2)	8 (5)	
Clinical T‐stage *n* (%)				0.012
1	64 (14)	49 (16)	15 (9)	
2	323 (68)	196 (64)	127 (75)	
3	88 (19)	62 (20)	26 (15)	
Clinical N‐stage *n* (%)				0.003
0	435 (91)	270 (88)	165 (98)	
1	21 (4)	19 (6)	2 (1)	
Missing	20 (4)	18 (6)	2 (1)	
EAU risk score *n* (%)				0.034
Low	27 (6)	17 (6)	10 (6)	
Intermediate	264 (55)	159 (52)	105 (62)	
High	169 (36)	123 (40)	46 (27)	
Missing	16 (3)	8 (3)	8 (5)	
Neoadjuvant ADT (*n* = 312) *n* (%)	10 (2)	7 (2)	3 (2)	0.854

*Note*: Percentages are presented as per their column.

Abbreviations: ADT, androgen deprivation therapy; ASA, American Society of Anaesthesia; EAU, European Association of Urology; ISUP, International Society of Urological Pathologists; IQR, interquartile range; PSA, prostate‐specific antigen.

### 3.3 Peri‐operative outcomes

Overall, the majority of RPs were performed robotically; however, there was more open surgery in the scheduled group compared to the delayed group (13% vs. 2%, *p* < 0.05) and subsequently less robotic‐assisted surgery (74% vs. 86%, *p* < 0.05). There was a greater proportion of trainee‐supervised operations as primary surgeon in scheduled versus delayed RP (18% vs. 7%, *p* < 0.05). The proportion of severe postoperative complications (Clavien‐Dindo ≥3) was higher in the scheduled group compared to the delayed group (5% vs. 2%, *p* < 0.05); however, there was a large proportion of missing data. Further peri‐operative data are displayed in Appendix A.

### 3.4 Oncological outcomes

The comparison of oncological characteristics between patients with scheduled and delayed RP is provided in Table 2. There was no statistically significant difference in the PSM rate between the two groups. Upstaging between the clinical T‐stage and specimen T‐stage was statistically significantly different between the groups. Forty‐nine percent of patients in the delayed group had upstaging compared to 35% of those in the scheduled group. There was no difference in upgradation from biopsy to specimen between the groups (*p* = 0.765).

**TABLE 2:** Postoperative oncological data.

	Overall	Time to surgery		
		Scheduled ≤90 days	Delayed >90 days	*p*‐Value
Positive surgical margins *n* (%)	137 (29)	91 (30)	46 (27)	0.512
Low risk	4 (15)	2 (12)	2 (20)	0.675
Intermediate risk	63 (24)	40 (25)	23 (22)	
High risk	69 (41)	48 (39)	21 (46)	
Missing	1 (0)	1 (0)	0 (0)	
Specimen T‐stage *n* (%)				
1	13 (3)	12 (4)	1 (1)	0.010
2	220 (46)	150 (49)	70 (41)	
3	235 (50)	138 (45)	97 (57)	
Missing	8 (2)	7 (2)	1 (1)	
Upstaging *n* (%)	189 (40)	107 (35)	82 (49)	0.008
Low risk	9 (33)	3 (18)	6 (60)	0.160
Intermediate risk	118 (45)	64 (40)	54 (51)	
High risk	57 (34)	38 (31)	19 (41)	
Missing	5 (31)	2 (25)	3 (38)	
Clinical N‐stage *n* (%)				
X/0	437 (92)	282 (92)	155 (92)	0.819
1	36 (8)	23 (7)	13 (8)	
Missing	3 (1)	2 (1)	1 (1)	
Specimen ISUP grade *n* (%)				
1	31 (7)	25 (8)	6 (4)	0.002
2	246 (52)	143 (47)	103 (61)	
3	134 (28)	85 (28)	49 (29)	
4	21 (4)	16 (5)	5 (3)	
5	36 (8)	31 (10)	5 (3)	
Missing	8 (2)	7 (2)	1 (1)	
Upgradation *n* (%)	85 (18)	57 (19)	28 (17)	0.765
Low risk	18 (67)	10 (59)	8 (80)	0.503
Intermediate risk	44 (17)	31 (19)	13 (12)	
High risk	23 (14)	16 (13)	7 (15)	
Biochemical recurrence	53 (11)	42 (14)	11 (7)	0.010
Low risk	1 (4)	0 (0)	1 (10)	0.143
Intermediate risk	26 (10)	21 (13)	5 (5)	
High risk	26 (15)	21 (17)	5 (11)	
Salvage ADT *n* (%)	41 (9)	33 (11)	8 (5)	0.081
Intermediate risk	13 (5)	11 (7)	2 (2)	0.552
High risk	25 (15)	19 (15)	6 (13)	
Missing	3 (19)	3 (38)	0 (0)	
Salvage radiotherapy	28 (6)	30 (10)	8 (5)	0.151
Intermediate risk	15 (6)	12 (8)	3 (3)	0.826
High risk	21 (12)	16 (13)	5 (11)	
Missing	2 (13)	2 (25)	0 (0)	

*Note*: Percentages are presented as per their column. Stratified risk group percentages are presented as a percentage of the overall number of sub‐group patients as per Table 1.

Abbreviations: ADT, androgen deprivation therapy; ISUP, International Society of Urological Pathologists.

Delay in surgery was not associated with PSM on univariable or multivariable analyses (adjusted for age, CCI score, time to surgery, ISUP grade, Clinical T‐stage, pre‐op PSA and prostate risk score) (Table 3). There was no difference in BCR between groups on adjusted multivariable analyses (Appendix B), and treatment allocation was not equal between the groups with the scheduled group including three times as many high‐risk patients as the delayed group.

**TABLE 3:** Logistic regression model for factors associated with positive surgical margins (yes/no).

	Univariable			Multivariable		
	Odds ratio (OR)	95% CI	*p*‐Value	Odds ratio (OR)	95% CI	*p*‐Value
Age (years)						
<50	Reference			Reference		
50–59	1.74	0.52–5.84	0.368	3.58	0.47–27.32	0.218
60–69	1.92	0.59–6.20	0.276	2.89	0.40–20.83	0.293
>70	2.27	0.67–7.69	0.188	3.46	0.45–26.78	0.234
CCI score						
0	Reference			Reference		
1	1.22	0.22–6.74	0.820	0.61	0.04–9.21	0.720
2	1.47	0.28–7.70	0.648	1.01	0.07–13.78	0.993
3	1.72	0.34–8.59	0.510	0.87	0.06–11.83	0.919
Time to surgery (days)						
≤90	Reference			Reference		
>90	1.00	0.62–1.61	0.996	1.07	0.63–1.81	0.801
ISUP grade						
1	Reference			Reference		
2	1.63	0.79–3.35	0.187	1.10	0.452–2.91	0.845
3	1.30	0.58–2.93	0.529	0.83	0.29–2.36	0.731
4	2.53	1.05–6.09	0.038	1.45	0.43–4.86	0.549
5	2.36	0.92–6.08	0.075	0.95	0.28–3.27	0.936
Clinical T‐stage						
0	Reference			Reference		
1	3.27	0.36–29.26	0.290	3.22	0.34–30.09	0.306
2	6.04	0.71–51.72	0.100	7.02	0.77–64.21	0.084
3	12.48	1.42–109.6	0.023	10.23	1.02–102.9	0.048
Pre‐op PSA	1.03	1.01–1.05	0.005	1.03	1.00–1.05	0.032
Risk score						
Low	Reference			Reference		
Intermediate	2.06	0.68–6.23	0.203	1.73	0.41–7.35	0.457
High	4.22	1.38–12.91	0.012	2.35	0.47–11.82	0.299

*Note*: Age, CCI score (Charlson co‐morbidity index), time to surgery, ISUP grade, clinical T‐stage, pre‐op PSA and risk score were pre‐specified variables.

Abbreviations: CI, confidence interval; ISUP, International Society of Urological Pathologists; PSA, prostate‐specific antigen.

## 4 DISCUSSION

During the pandemic, high‐risk patients were prioritised for PCa surgery across the world. We found that early oncological outcomes were not adversely impacted by delaying RP by more than 90 days after the MDT decision date to operate for across all risk groups.

Previous retrospective and national cancer‐registry studies suggest that delay to RP is safe with no associated adverse oncological outcomes.^16^, ^17^ Ginsburg et al. reviewed over 100 000 men with registry data and found no difference in treatment failure rates between patients undergoing RP within 3 months compared to RP up to 12 months after diagnosis.^6^ However, there may be selection bias in this data due to the inclusion of men who initially underwent active surveillance. Laukhtina et al. found no difference in oncological outcomes if surgery was delayed for 3 months.^18^ Further systematic reviews by Chan et al. struggled to draw conclusions due to the heterogenicity in study designs.^19^ Our study uses a unique methodology brought about by the COVID‐19 pandemic—It is the first prospective, international study observing patients due RP at the time of cancer diagnosis. Despite the difference in methodology, our study also shows no difference in the PSM rate or BCR between delayed and scheduled surgery, across risk groups, when other key variables are accounted for.

Conversely, Berg et al. reported adverse pathological outcomes when RP is delayed by 30–60 days for intermediate‐ and high‐risk disease.^4^ However, the study used stratification rather than multivariable analysis, due to the limited sample size, and could not adjust for all confounders. A larger, population‐based study using Swedish cancer data found men waiting more than 2 years for RP were twice as likely to need further salvage treatment, but there was no difference in cancer‐specific mortality at a median follow‐up of 8.1 years.^20^ Despite resource, pandemic or time to decision‐making pressures, it is clinically unlikely a patient would need to wait longer than 2 years for a RP. In our study, we did find an increase in upstaging in the delayed group compared to the scheduled group, which is associated with inferior longer term oncological outcomes.^21^ However, due to the limited follow‐up of our study, our main limitation, we cannot present data on the implications of the increased upstaging observed. A further limitation to our study is the heterogeneity due to a multi‐national dataset, which provides poor precision in estimating the risk factors associated with oncological outcomes.

Our results add to the literature in terms of early surrogate markers of oncological outcomes, such as PSM and upgradation rates, and our study is unique in terms of its prospective data as a result of the COVID‐19 pandemic.

In accordance with NCCN guidelines and consensus at the time of the pandemic, patients with high‐risk disease were prioritised for RP. Only 27% of our delayed cohort were high risk compared to 40% of the scheduled cohort. Surprisingly, despite respiratory and mortality concerns of surgery during the pandemic, a greater proportion of more co‐morbid, less fit men underwent scheduled rather than delayed surgery, suggesting that risk stratification was prioritised over co‐morbidities.^22^

In conclusion, our study found no significant differences in early oncological outcomes for men undergoing RP more than 90 days compared to men undergoing RP within 90 days after the decision to treat. The decisions made to delay surgery during the COVID‐19 pandemic do not appear to have adversely impacted short‐term oncological outcomes in this patient cohort. The implications of this may be better resource planning for hospitals whilst patients are allowed a greater amount of time to consider various treatment options available to them.

## AUTHOR CONTRIBUTIONS

*Study design*: Arjun Nathan, Chuanyu Gao, Cameron Alexander, Alexander Light, Vinson Chan, Kevin Gallagher, Sinan Khadhouri, Alan McNeill, Krishna Narahari, Grant D. Stewart and Veeru Kasivisvanathan. *Data analysis*: Arjun Nathan and Kelvin Okoth. *Article draft*: Arjun Nathan, Cameron Alexander, Alexander Light and Sinan Khadhouri. *Critical revision*: Alan McNeill, Krishna Narahari, Grant D. Stewart and Veeru Kasivisvanathan. *Publication approval*: All authors.

## CONFLICT OF INTEREST STATEMENT

All other authors declare no conflict of interest.

## Appendix A OPERATIVE DATA

**Table:**

	Overall	Time to surgery		
		Scheduled ≤90 days	Delayed >90 days	*p*‐Value
Type of operation *n* (%)				0.001
Open	44 (9)	40 (13)	4 (2)	
Laparoscopic	57 (12)	37 (12)	20 (12)	
Robotic	375 (79)	230 (74)	145 (86)	
Console time (minutes) (*n* = 185) median (IQR)	125 (90–15)	130 (99–175)	125 (90–15)	0.433
EBL (millilitres) (*n* = 464) median (IQR)	150 (50–250)	150 (50–300)	100 (50–250)	0.323
Pelvic lymph node dissection *n* (%)	235 (49)	158 (51)	77 (45)	0.201
Intra‐operative complication (*n* = 33) *n* (%)	1 (3)	0 (0)	1 (1)	0.632
Operation performed by *n* (%)				0.001
Consultant	409 (86)	252 (82)	157 (93)	
Trainee supervised	67 (14)	55 (18)	12 (7)	
Clavien‐Dindo complication *n* (%)				0.001
≥III	17 (4)	14 (5)	3 (2)	
Missing	48 (10)	15 (5)	33 (20)	
30‐Day postoperative complication *n* (%)				
Anastomotic leak	19 (4)	15 (5)	4 (4)	0.179
UTI	29 (6)	22 (7)	7 (4)	0.187
Haematoma	12 (3)	9 (3)	3 (2)	0.706
Ileus	1 (0)	0 (0)	1 (1)	0.051
Bowel injury	0 (0)	0 (0)	0 (0)	0.952
Wound infection	8 (2)	7 (2)	1 (1)	0.522
VTE	5 (1)	4 (1)	1 (1)	0.952
Haematuria	6 (1)	5 (2)	1 (1)	0.781
Other	24 (5)	20 (7)	4 (2)	0.489

Abbreviations: EBL, estimated blood loss; IQR, interquartile range; UTI, urinary tract infection; VTE, venous thromboembolism.

## Appendix B LOGISTIC REGRESSION MODEL FOR FACTORS ASSOCIATED WITH BIOCHEMICAL RECURRENCE (YES/NO)

**Table:**

	Univariable			Multivariable		
	Odds ratio (OR)	95% CI	*p*‐Value	Odds ratio (OR)	95% CI	*p*‐Value
Age (years)						
<50	Reference			Reference		
50–59	0.58	0.05–6.36	0.657	0.66	0.07–6.13	0.719
60–69	0.26	0.02–3.19	0.292	0.31	0.03–3.10	0.320
>70	0.36	0.03–4.58	0.427	0.45	0.04–4.87	0.511
CCI score						
0	Reference			Reference		
1	0.84	0.03–25.90	0.918	1.00	0.04–26.32	0.999
2	2.30	0.07–76.80	0.641	2.38	0.09–63.63	0.604
3	2.98	0.09–95.61	0.537	3.34	0.13–88.22	0.471
Time to surgery (days)						
≤90	Reference			Reference		
>90	0.79	0.32–1.98	0.620	0.61	0.26–1.41	0.248
ISUP grade						
1	Reference			Reference		
2	0.33	0.08–1.40	0.136	0.37	0.09–1.52	0.169
3	0.48	0.11–2.13	0.335	0.62	0.14–2.64	0.517
4	0.62	0.11–3.42	0.584	0.73	0.13–3.99	0.714
5	2.34	0.41–13.20	0.336	2.77	0.50–15.50	0.246
Clinical T‐stage						
0/1	Reference			Reference		
2/3	1.52	0.42–5.56	0.523	1.46	0.40–5.33	0.567
Pre‐op PSA	0.99	0.96–1.03	0.763	1.03	1.00–1.05	0.032
Risk score						
Low	Reference			Reference		
Intermediate	7.80	0.7–86.85	0.095	7.39	0.67–82.02	0.103
High	8.34	0.66–104.9	0.101	7.94	0.63–100.5	0.110

*Note*: Age, CCI score (Charlson co‐morbidity index), time to surgery, ISUP grade, clinical T‐stage, pre‐op PSA and risk score were pre‐specified variables.

Abbreviations: CI, confidence interval; ISUP, International Society of Urological Pathologists; PSA, prostate‐specific antigen.
